# Anemia and other hematological profiles of pregnant women attending antenatal care in Debre Berhan Referral Hospital, North Shoa, Ethiopia

**DOI:** 10.1186/s13104-018-3805-8

**Published:** 2018-10-05

**Authors:** Dessalegn Shitie, Tewabech Zewde, Yalew Molla

**Affiliations:** 1 Biomedical Science Department, School of Medicine, Debre Markos University, P. O. Box, 269, Debre Markos, Ethiopia; 2 Physiology Department, Addis Ababa University, P. O. Box, 1176, Addis Ababa, Ethiopia; 3 Pharmacy Department, College of Health Sciences, Debre Markos University, P. O. Box, 269, Debre Markos, Ethiopia

**Keywords:** Anemia, Pregnancy, Antenatal care, Trimester, Hematological profiles

## Abstract

**Objective:**

The aim of the study was to determine level of anemia and other hematological profiles in pregnant women attending antenatal care clinic in Debre Berhan Referral Hospital, Ethiopia.

**Results:**

Prevalence of anemia was 2.8% and that of thrombocytopenia was 10.2%. Out of the anemic pregnant mothers, 5 (62.5%) were mildly anemic and 2 (25%) were severely anemic. The factor age < 20 years of mothers was significantly associated with anemia (P < 0.05). In addition, the occurrence of anemia in mothers who visited antenatal clinic two times is two times higher than those mother who visited the antenatal clinic three times. Moreover, the prevalence of anemia is two times more likely to occur in pregnant mothers who did not take iron supplements as compared to their counter parts. According to pregnancy periods; mean white blood cells count was (8.48 ± 3.09, 8.83 ± 2.73, 8.86 ± 2.67) × 10^9^/L for the first, second and third trimesters, respectively. Red blood cells and platelet counts in the first trimester were significantly higher than their corresponding values in third trimester (P < 0.01), whereas mean hemoglobin and hematocrit values were not statistically significant within trimesters (P > 0.05).

**Electronic supplementary material:**

The online version of this article (10.1186/s13104-018-3805-8) contains supplementary material, which is available to authorized users.

## Introduction

Change in hematological profiles is one of the factors affecting pregnancy and its outcome. As a result, hematological profiles are measured all over the world to estimate general health status of individuals because of its reliability and cost-effectiveness [1]. Severe anemia during pregnancy is associated with prematurity, spontaneous abortions, low birth weight, operative delivery, postpartum hemorrhage and fetal death [1, 2].

Anemia is the most common hematological problem during pregnancy followed by that of thrombocytopenia [1, 3]. The global prevalence of anemia in pregnant women is 41.8%. In Africa, the prevalence of anemia is 55.8% which is comparably greater than its prevalence in Asia (41.6%), and Europe (18.7%) [3]. These figures reflect that the burden of anemia is more in pregnant women living in the poorest region of the world.

The common causes of anemia are malnutrition, pregnancy, infections with malaria, hook worm, HIV, haemoglobinopathies and obstetrical complications causing abnormal blood loss [4, 5]. Socioeconomic status of the family, traditional eating habits of the region, fear of weight gain and irregular eating habits are major behavioral risk factors for the development of anemia in adolescent [6].

In addition, family illiteracy, malaria, and duodenal ulcer bleeding are predisposing factors of anemia in pregnant women [7]. Moreover, low family income, high family size, living with HIV/AIDS, residence in rural area, intestinal parasitic infection, walking bare foot, and history of heavy menstrual cycle (> 5 days of menses) are known to be the predictors of anemia among pregnant women in Ethiopia [8, 9]. Despite its decline through time, anemia is still the major health problem in women of reproductive age [10].

Even though various studies have been conducted in developed countries to determine the hematological profiles of pregnant women, little is known in developing countries like Ethiopia, particularly in the selected study area. Thus, purpose of this study was to determine the prevalence of anemia and other hematological profiles and associated factors among pregnant women attending antenatal care clinic in Debre Berhan Referral Hospital (DBRH), Ethiopia.

## Main text

### Study design, period and setting

Institution based cross sectional study design was employed in pregnant women who visited antenatal care (ANC) clinic at Debre Berhan Referral Hospital (DBRH). The study was conducted from October to January 2015 GC.

The study was conducted in DBRH which is found in Debre Berhan town located in the North East of Addis Ababa at a distance of 123 Kms. The hospital provides ANC services together with other maternal and child health care and medical services.

### Sample size determination

Actual sample size for the study was determined using the formula for single population proportion by assuming 5% margin of error and 95% confidence interval (α = 0.05) and using the previous prevalence of 21.3% [11]. The actual sample size with 10% contingency was 284.

### Sample recruitment and sampling procedure

All pregnant women who came to DBRH for ANC in maternal and child health department were screened for eligibility in the study. Pregnant women who were sick during data collection, those having bleeding in the pregnancy, recently transfused, having known chronic diseases and diagnosed with haemoglobinopathies were excluded. Based on the previous 3 months record, the target pregnant mothers were selected by systematic sampling technique at a sampling interval of three. The first interviewed participant was randomly selected by a lottery system.

### Data collection procedure

Data on socio-demographic variables and obstetric characteristics were collected by trained nurses using structured interviewer administered questionnaire. Venous blood was collected once from each pregnant women as they visit the ANC clinic during the data collection period regardless of their gestational age by well-trained laboratory technologists. The collected venous blood was tested in DBRH hematology laboratory and results were collected timely.

### Data analysis

Data were entered into Epi-Info version 3.1 and analyzed using SPSS version 21. Bivariate logistic regression was computed to assess statistical association between anemia and independent variables. Significance of statistical association were tested at P-value < 0.05. After bivariate logistic regression analysis, variables with P-value < 0.3 were entered to multivariate logistic regression analysis. The Mann–Whitney U test was utilized to compare mean of hematological values in different trimesters.

### Results

#### Hematological profiles of pregnant women

Overall hematological parameters of 284 pregnant women were described as mean ± SD. Among the pregnant women, 46, 100, 138 were in the first, second and third gestational age (Additional file 1: Table S2). White blood cells (WBC) and neutrophil count were (8.79 ± 2.76 and 6.14 ± 2.41) × 10^9^/L, respectively. Red blood cells (RBC) count (4.53 ± 0.73 × 10^12^/L), hemoglobin (14.89 ± 2.45 g/dL), hematocrit (Hct) (42.50 ± 6.59%), and platelet (PLT) count (242.85 ± 83.90 × 10^9^/L) were recorded in pregnant women participated in the study (Table 1).

**Table 1:** Overall and trimester based hematological profiles of pregnant women (mean ± SD) in Debre Berhan Referral Hospital, North Shoa, Ethiopia, October to January 2015

Complete blood count results	Over all	Trimesters (number of women)			P-value		
		1st (n = 46)	2nd (n = 100)	3rd (n = 138)	1st and 2nd	1st and 3rd	2nd and 3rd
WBCs, × 10^9^/L	8.79 ± 2.76	8.48 ± 3.09	8.83 ± 2.73	8.86 ± 2.67	0.34	0.31	1.00
Differential WBC count in #							
Neutrophils, × 10^9^/L	6.14 ± 2.41	5.56 ± 2.55	6.23 ± 2.48	6.26 ± 2.30	0.14	0.08	0.89
Lymphocytes, × 10^9^/L	2.02 ± 0.63	2.28 ± 0.75	1.99 ± 0.60	1.96 ± 0.6	0.040*	0.01*	0.63
Mid WBCs, × 10^9^/L	0.62 ± 0.25	0.61 ± 0.29	0.62 ± 0.23	0.63 ± 0.25	0.569	0.44	0.78
Differential WBC count in %							
Neutrophils	68.06 ± 9.80	63.75 ± 10.14	68.07 ± 11.42	69.47 ± 7.89	0.01**	0.000**	0.67
Lymphocytes	24.40 ± 8.36	28.83 ± 9.71	24.08 ± 8.66	23.16 ± 7.14	0.002**	0.000**	0.74
Mid	7.37 ± 2.09	7.29 ± 2.14	7.42 ± 2.12	7.36 ± 2.08	0.98	0.868	0.85
RBCs, × 10^12^/L	4.53 ± 0.73	4.68 ± 0.52	4.55 ± 0.82	4.48 ± 0.71	0.051	0.002**	0.16
Hemoglobin (g/dL)	14.89 ± 2.45	15.06 ± 1.70	14.68 ± 2.56	14.99 ± 2.58	0.41	0.441	0.767
Hematocrit (%)	42.50 ±6.59	42.88 ± 4.62	42.08 ± 6.46	42.68 ± 7.23	0.35	0.34	0.99
MCV (fL)	94.07 ± 6.99	91.93 ± 3.83	93.46 ± 6.75	95.23 ± 7.76	0.012*	0.000**	0.02*
MCH (pg)	33.08 ± 2.48	32.3 ± 1.44	32.79 ± 2.82	33.55 ± 2.41	0.02*	0.000**	0.03*
MCHC (g/dL)	35.10 ± 1.09	35.12 ± 0.95	35.09 ± 1.26	35.09 ± 1.03	0.76	0.99	0.78
RDW (%)	13.92 ±2.17	13.92 ± 3.58	13.78 ± 1.42	14.03 ± 2.01	0.08	0.02*	0.38
Platelet count (× 10^9^/L)	242.85 ± 83.90	272.91 ± 89.17	253.19 ± 96.14	225.33 ± 67.53	0.09	0.001**	0.057
MPV (fL)	8.45 ± 0.89	8.45 ± 0.84	8.36 ± 0.86	8.52 ± 0.94	0.42	0.85	0.236

*n* number of women in each trimester, *Hct* hematocrit, *Hgb* hemoglobin, *MCH* mean corpuscular hemoglobin, *MCHC* mean corpuscular hemoglobin concentration, *MCV* mean corpuscular volume, *PLT* platelet, *RBC* red blood cell, *RDW* red cell distribution width

* P-value < 0.05, ** P-value < 0.01

According to pregnancy periods; mean WBCs count has been shown to increase with gestational age. RBCs and platelet counts in the first trimester were significantly higher than their corresponding values in third trimester (P < 0.01), whereas mean hemoglobin and hematocrit values were not statistically significant within trimesters (P > 0.05). The mean MCV and MCH values had been shown to increase with the advancement of pregnancy period. There was significant MCV differences between first and second trimesters, second and third trimesters, and first and third trimesters of pregnant women (P = 0.012, 0.02, and 0.00, respectively). In addition, there was also significant MCH differences between first and second trimesters, second and third trimesters, and first and third trimesters of pregnant women (P = 0.02, 0.03, and 0.00, respectively) (Table 1).

#### Prevalence of anemia and thrombocytopenia

Based on WHO criteria, hemoglobin value of less than 11.0 g/dL is considered as indicative of anemia for pregnant women. Accordingly, in the present study, 8 (2.8%) of study participants were anemic. Out of 284 pregnant women, 29 were thrombocytopenic (Platelet count < 150 × 10^9^/L) with a prevalence of 10.2%. Out of the anemic pregnant mothers, 5 (62.5%) were mildly anemic and 2 (25%) were severely anemic (Table 2).

**Table 2:** Distribution of anemia and thrombocytopenia by severity among anemic and thrombocytopenic pregnant women at Debre Berhan Referral Hospital, North Shoa, Ethiopia, October to January 2015

Severity	Anemia number (%)	Thrombocytopenia number (%)
Mild	5 (62.5)	22 (75.86%)
Moderate	1 (12.5)	6 (20.69%)
Severe	2 (25)	1 (3.45%)

(n = 8)

#### Bivariate and multivariate analysis

Prevalence of anemia among pregnant women with age groups of less than 20 years were 29.5 times higher than those with age groups of greater or equals to 26 years. The occurrence of anemia in mothers who visited ANC clinic two times is two times higher than those mothers who visited the ANC clinic three or more times. In addition, the prevalence of anemia is two times more likely to occur in pregnant mothers who did not take iron supplements as compared to their counter parts (Table 3). However, by adjusting potential confounders in multivariate logistic regression analysis, only age groups < 20 years old was significantly associated with anemia (P < 0.05) (Table 3).

**Table 3:** Prevalence of anemia by socio-demographic, obstetric and other characteristics of pregnant women at Debre Berhan Referral Hospital, North Shoa, Ethiopia, October to January 2015

Variables	Anemic status		COR (95% CI)	AOR (95% CI)
	Anemic (%)	Non anemic (%)		
Age				
< 20	5 (11.6%)	38 (88.4%)	*17.6 (1.99, 155.5)*	29.5 (1.99, 436.7)*
20–25	2 (1.9%)	104 (98.1%)	2.58 (0.231, 28.8)	5.24 (0.35, 78.8)
≥ 26	1 (0.74%)	134 (99.26%)	1	1
BMI				
< 19.8	2 (8.3%)	22 (81.7%)	3.8 (0.73, 20.2)	4.15 (0.55, 31.2)
≥ 19.8	6 (2.3%)	254 (97.7%)	1	1
Marital status				
Single	1 (11.1%)	8 (89.9%)	4.79 (0.53, 43.6)	5.86 (0.43, 78.8)
Married	7 (2.5%)	268 (97.5%)	1	1
Residence				
Urban	5 (2.3%)	209 (97.7%)	0.53 (0.12, 2.3)	0.66 (0.09, 4.86)
Rural	3 (4.3%)	67 (95.7%)	1	1
Educational status				
Illiterate	1 (1.8%)	54 (98.2%)	0.82 (0.26, 4.7)	0.62 (0.03, 11.59)
Primary	3 (6.1%)	46 (93.9%)	2.9 (0.62, 13.3)	2.87 (0.35, 23.61)
Secondary and above	4 (2.2%)	176 (97.8%)	1	1
Gestational age				
1st trimester	0	46 (100%)	000	000
2nd trimester	5 (5%)	95 (95%)	2.37 (0.55, 10.2)	1.49 (0.26, 8.64)
3rd trimester	3 (2.2%)	135 (97.8%)	1	1
Parity				
Para 0	5 (3.3%)	146 (96.7%)	1.48 (0.35, 6.33)	0.34 (0.05, 2.46)
Para 1+	3 (2.3%)	130 (97.7%)	1	1
Age of last child				
≤ 2	1 (5.3%)	18 (94.7%)	3.2 (0.28, 37.6)	
> 2	2 (1.8%)	112 (98.2%)	1	
Number of ANC visit				
1	3 (2.6%)	111 (97.4%)	1.2 (0.19, 7.27)	1.18 (0.06, 23.64)
2	3 (3.8%)	77 (96.2%)	1.7 (0.28, 10.5)	1.99 (0.21, 18.39)
3+	2 (2.2%)	88 (97.8%)	1	1
Taking iron supplements				
Yes	5 (2.9%)	166 (97.1%)	1.1 (0.26, 4.7)	0.72 (0.06, 8.29)
No	3 (2.7%)	110 (97.3%)	1	1

Italic data denote some significances

(N* = *284)

*ANC* antenatal care, *BMI* body mass index

* P-value < 0.05

### Discussion

Monitoring hematological profiles is essential to diagnose or monitor illness in pregnant woman. Prevalence of anemia in this study is very low compared with the findings of various studies conducted in Ethiopia and foreign countries. For instance, the prevalence of anemia in India, Nigeria, Kenya, Mexico and other regions of Ethiopia [8, 9, 11–20] was higher than that of the finding of the present study. This discrepancy might be due to difference in socioeconomic and educational status, food selection habits, multi-factorial causes of anemia, prevalence of malaria and other intestinal parasites in some study area, access to health care services, access to iron supplementation, awareness in pregnant women to ANC follow and methods used to determine hemoglobin where some might be exposed to bias compared with automated hematology analyzers used in the present study.

In addition, the low prevalence of anemia observed in the present study might also be due to the high altitude (2780 m) and low prevalence of malaria of the study area [21]. Moreover, as indicated on the sociodemographic data (Additional file 1: Table S1), majority of participants were urban resident and wearing shoes which prevents the participants from being affected by parasites which could in turn affect the level of RBC of participants.

Even though the overall anemia prevalence is low in this study, the level of severe anemia was high compared with previous studies in Bahir Dar [20], south east Ethiopia [16], Gilgel Gibe dam area [9], Kenya [14] and in Addis Ababa [11]. The reasons for the differences might be associated with differences in socioeconomic and awareness of pregnant women to symptoms and outcomes of anemia on maternal and fetal health among the target regions and countries.

According to the present study, pregnant woman with age groups < 20 years old were 29.5 times more likely to develop anemia than age groups > 26 years old. This finding was consistent with studies in Kenya where low age was more likely to develop anemia [14]; but did not agree with studies in Addis Ababa where higher age groups [39–45 years] had significant association with anemia [11]. This discrepancy between the findings of these studies and the present study might be due to difference in time of study, life style and access to health care facilities among study participants. Although other variables were not significantly associated with anemia, in the present study, low BMI, being in second trimester gestational status, and low frequency of ANC follow up were the factors that prone women to develop anemia. Whereas urban residence, having secondary and above educational level, being nulliparous, women with iron supplement were the less likely associated factors to develop anemia.

Prevalence of thrombocytopenia was high in third trimester. This finding is relatively consistent with studies in Iraqi [22, 23] and India [24, 25]. In this study, the platelet count was much lower in third trimester than that in the first and second trimester as that of the findings obtained in studies conducted in Lagos, Nigeria, Abia State, Nigeria and Jamaica [1, 26, 27]. The lower platelet count with gestational age might be due to hemodilution secondary to expansion of plasma volume during pregnancy. The declining trend of platelet with increasing gestational age predispose pregnant women to risk of hemorrhage [25]. Thus, platelet count should be routinely performed during antenatal visit for timely diagnosis and to obtain good feto-maternal outcome in all types of thrombocytopenia during pregnancy.

The mean total WBCs count progressively increased from first to third pregnancy period. This might be due to physiologic stress induced by pregnancy, and the needs of the developing fetus. The increased WBCs count with gestational age was in line with the finding of a study done in Port Harcourt [27]. However, the present finding is inconsistent with study in Nigeria and Jamica where WBCs count significantly varied between trimesters [26, 27]. The discrepancy might be because of time, life style and population differences between study groups.

RBCs count was significantly higher in the first trimester compared with the third (4.68 ± 0.52 versus 4.48 ± 0.71, P < 0.01) but changes in Hgb and Hct values were not significant between all the trimesters. This finding did not agree with the findings obtained in studies conducted in Port Harcourt and Nigeria, where Hct values significantly decreased as gestational age increases [1, 28]. A study conducted in Nigeria indicated that most RBC indices were significantly changed across trimesters [11]. Moreover, a study in Jamaica revealed that RBCs count significantly changed between 1st–2nd and 2nd–3rd trimesters [27]. These discrepancies might be due to geographical, educational, economical, health care facility and behavioral differences among study areas.

MCV and MCH values significantly increased from first to third trimester while MCHC was relatively constant in the present study. This is relatively consistent with a study in Jamaica [27]. However, a study done in Nigeria revealed that MCV declined from first to third trimesters, MCH remained relatively stable through all trimesters and MCHC was stable in the first and second trimester but dropped in third [1]. This might reflect the difference in existence of iron deficiency among the study participants of the studied countries. In addition, the increased level of MCV and MCH with gestational age might be associated with the low prevalence of anemia in the participants and that of well controlled supply of micronutrients like iron for the normal maintenance of hematologic profiles of the majority of the study participants of this study relative to the dilution effect of their plasma volume.

### Conclusions

The prevalence of anemia is more common in pregnant mothers with younger age, in those who have relatively less frequent ANC follow up and in those who do not take iron supplements regularly. There are variations of the count of RBCs, MCV, MCH and platelets values during the whole trimesters of pregnant mothers. Therefore, complete blood count has to be done to diagnose and prevent the potential development of anemia, thrombocytopenia and other hematologic abnormalities in pregnant mothers during antenatal care.

## Limitations

The study did not use cohort techniques to assess the hematologic profiles of the pregnant mothers across the gestational period because of time and budget constraints.

## Additional file

**Additional file 1.** Socio-demographic characteristic of pregnanat women at Debre Birhan Referral Hospital, North Shoa, Ethiopia, October to January, 2015.

## Abbreviations

AAU: Addis Ababa University
ANC: antenatal care
DBRH: Debre Berhan Referral Hospital
Hct: hematocrit
Hgb: hemoglobin
MCH: mean corpuscular hemoglobin
MCHC: mean corpuscular hemoglobin concentration
MCV: mean corpuscular volume
PLT: platelet
RBC: red blood cell
RDW: red cell distribution width
SPSS: Statistical Package for Social Sciences
WBC: white blood cell
WHO: World Health Organization
