# Supplementary material for: Anemia and other hematological profiles of pregnant women attending antenatal care in Debre Berhan Referral Hospital, North Shoa, Ethiopia
Source: BMC Res Notes. 2018 Oct 5;11:704. doi: 10.1186/s13104-018-3805-8 (PMC6173918; doi:10.1186/s13104-018-3805-8)
Supplement: Supplementary file 1 — Additional file 1. Socio-demographic characteristic of pregnanat women at Debre Birhan Referral Hospital, North Shoa, Ethiopia, October to January, 2015. [file 13104_2018_3805_MOESM1_ESM.docx]

# Additional files

# Socio demographic characteristics of pregnant women

The mean age of participants was 25.95 + 5.11 years. Majority of pregnant women, 219 (77.1%) had BMI ranges from 19.8-25.99 kg/m^2^. Most study participants were married 275 (96.8%), urban resident 214 (75.4%), in secondary school education level 101 (35.6%) and orthodox religion followers 274 (96.5%) (Table S1).

Table S1. Socio-demographic characteristics of pregnant women at Debre Berhan Referral Hospital, North Shoa, Ethiopia, October to January 2015. (N=284)

| Variables | Frequency | Percentage (%) |
| --- | --- | --- |
| **Age(years)**  <20  20-25  26-30  31-35  >35 | 43  106  103  16  16 | 15.2  37.3  36.3  5.6  5.6 |
| **Body mass index(kg/m^2^)**  <19.8  19.8-25.99  26-29  >29 | 24  219  36  5 | 8.4  77.1  12.7  1.8 |
| **Marital status**  single  Married | 9  275 | 3.2  96.8 |
| **Ethnicity**  Amhara  Oromo | 278  6 | 97.9  2.1 |
| **Residence**  urban  Rural | 214  70 | 75.4  24.6 |
| **Educational status**  Illiterate  Primary  Secondary  Higher | 55  49  101  79 | 19.4  17.2  35.6  27.8 |
| **Religion**  Orthodox  Muslim  Protestant | 274  9  1 | 96.5  3.2  0.3 |

# Obstetric characteristics of pregnant women

Among the study participants, 46(16.2%), 100(35.2%) and 138(48.6%) were in their first, second and third trimester, respectively. Based on some obstetric history of pregnant women: 68 (23.9%) had their first delivery when they were 21-25 years old, 151 (53.2%) primigravidae, 127(44.7%) had > 2 years interval between current pregnancy and last child, 19(6.7%) had history of abortion, 114(40.1%) visited ANC clinic once while 25(8.8%) visited four times. Almost all study participants didn^’^t take anthelminthic and antimalarial drugs, did not have history of recent blood transfusion and bleeding in this pregnancy and had no history of smoking cigarette and drinking alcohol. Among all participants, 161(94.2%) took iron supplements for three months (Table S2).

Table S2. Obstetric characteristics of pregnant women at Debre Berhan Referral Hospital, North Shoa, Ethiopia, October to January 2015. (N = 284)

| Variables | Frequency | Percentage |
| --- | --- | --- |
| **Gestational age**  First trimester  Second trimester  Third trimester | 46  100  138 | 16.2  35.2  48.6 |
| **Age at first delivery(years)**  Not delivered (primigravidae)  < 15  15- 20  21-25  > 25 | 151  1  57  68  7 | 53.2  .3  20.1  23.9  2.5 |
| **Parity**  0  1  2  >= 3 | 151  65  37  31 | 53.2  22.9  13.0  10.9 |
| **Age of last child(year)**  None  1  2  3  4 | 151  6  13  47  67 | 53.2  2.1  4.6  16.5  23.6 |
| **Abortion** | 19 | 6.7 |
| **Those got medical care** | 19 | 100.0 |
| **Place of medical care**  Health post  Health center  Hospital | 1  4  14 | 5.3  21  73.7 |
| Number of ANC visit  1  2  3  4 | 114  80  65  25 | 40.1  28.2  22.9  8.8 |
| Taking iron supplements | 171 | 60.2 |
| Duration of iron supplements in months  1  2  3 | 6  4  161 | 3.5  2.3  94.2 |

Based on red blood cell morphologic classification of anemia, most of the anemic pregnant women had microcytic hypochromic 5(62.5 %) type of anemia (Figure 1).

Figure S1. Distribution of morphologic type of anemia among anemic pregnant women at Debre Berhan Referral Hospital, North Shoa, Ethiopia, October to January 2015. (n= 8**)**
